# Molecular study of lysosomal storage disorders in India

**DOI:** 10.1186/1755-8166-7-S1-I30

**Published:** 2014-01-21

**Authors:** Jayesh Sheth

**Affiliations:** 1 FRIGE’s Institute of Human Genetics, FRIGE House, Satellite, Ahmedabad, India

## 

Lysosomal storage disorders (LSDs) are gaining greater attention of the researcher and medical fraternity due to increasing awareness of occurrence, diagnostic facility, prenatal diagnosis and options for therapeutic intervention. Though the exact incidence is not known, they are likely to occur with a ratio ranging from 1:6000-1:8000 with the highest occurrence of Gaucher disease followed by GM2 gangliosidosis and mucopolysachharide disorder. However, our knowledge about the mutation spectrum for most of the LSDs and its phenotype consequences is limited.

The present study was aimed to identify disease causing mutation for *HEXA* and *GBA* gene and its phenotypic consequences.

Study was carried out in 37 biochemically confirmed subjects having Tay-Sachs disease and 32 subjects with Gaucher disease. Molecular analysis was carried out for all exons and exon-intron boundaries of *HEXA* and *GBA* gene by bidirectional sequencing method. *In silico* analysis was carried out using SIFT, Polyphen2 and Mutation T@ster softwares.

In *HEXA* gene, 21 mutations were identified in 34 unrelated families in Tay-Sachs disease, 15 of which were novel, including 10 missense mutations [E114K, D175A, T263I, G269R, D322N, D322Y, Q374P, R393P, E462V, G478R] in 20 families, 4 nonsense mutations [Q237X, W474X, W485X, R510X] in 5 families, one 8 bp deletion [c.898_905TTCATGAG (p.F300HfsX21)] in one family. Earlier known 6 mutations were also observed that include 2 missense mutations [R170W and R178C] in 2 patients, one 4bp insertion in 5 patients [c.1277_1278insTATC (p.Y427IfsX5)], 3 splice site mutations [c.805+1 G>C, c.459+5 G>A and c.672+30 T>G] in four families. Mutation was not identified in 3 families. In *GBA* gene (Gaucher disease) we have identified 10 missense mutations in 32 unrelated families that include 9 known mutations [E326K, V352M, G355D, S356F, R359Q, R368C, R395C, R463C and L444P] in 31 families and one novel mutation [G289A] in one family. *In silico* analysis further confirmed the pathogenic effect of the novel mutations occurred at highly evolutionarily conserved and functionally active domain residues in the protein leading to conformational changes or mRNA producing truncated protein resulting in the diminish or absent activity of the protein.

Present study demonstrated that E462V, D322Y and c.1277_1278insTATC (p.Y427IfsX5) mutations are the most common mutations observed in nearly 49% (18/37) of the children with TSD in India. Similarly, L444P missense mutation is commonly observed in 21/32 (65.62%) children with Gaucher disease. Additionally, the data also demonstrates that exon 5-12 in *HEXA* gene and exon 8-10 in GBA gene are the hot spot region where ~75% and 94 % mutations can be identified in Indian patients with the aforementioned diseases respectively.

